# Error Analysis of Accelerometer- and Magnetometer-Based Stationary Alignment

**DOI:** 10.3390/s21062040

**Published:** 2021-03-14

**Authors:** Felipe O. Silva, Lucas P. S. Paiva, Gustavo S. Carvalho

**Affiliations:** Department of Automatics, Federal University of Lavras, Lavras 37200-900, Brazil; lucas.paiva1@estudante.ufla.br (L.P.S.P.); gustavo.carvalho8@estudante.ufla.br (G.S.C.)

**Keywords:** navigation, AHRS, accelerometer, magnetometer, stationary alignment, error analysis

## Abstract

This paper revisits the stationary attitude initialization problem, i.e., the stationary alignment, of Attitude and Heading Reference Systems (AHRSs). A detailed and comprehensive error analysis is proposed for four of the most representative accelerometer- and magnetometer-based stationary attitude determination methods, namely, the Three-Axis Attitude Determination (TRIAD), the QUaternion ESTimator (QUEST), the Factored Quaternion Algorithm (FQA), and the Arc-TANgent (ATAN). For the purpose of the error analysis, constant biases in the accelerometer and magnetometer measurements are considered (encompassing, hence, the effect of hard-iron magnetism), in addition to systematic errors in the local gravity and Earth magnetic field models (flux density magnitude, declination angle, and inclination angle). The contributions of this paper are novel closed-form formulae for the residual errors (normality, orthogonality, and alignment errors) developed in the computed Direction Cosine Matrices (DCM). As a consequence, analytical insight is provided into the problem, allowing us to properly compare the performance of the investigated alignment formulations (in terms of ultimate accuracy), as well as to remove some misleading conclusions reported in previous works. The adequacy of the proposed error analysis is validated through simulation and experimental results.

## 1. Introduction

Inertial Navigation Systems (INSs) are specialized dead-reckoning systems which provide a standalone navigation solution for attitude, velocity, and position [1]. Like any dead-reckoning system, INSs need to comply with a generally stationary initialization procedure, which, in the specific case of the attitude, is called alignment [2,3]. The very purpose of the alignment is to roughly estimate the attitude of the vehicle (or body) frame relative to the navigation frame, so that it can be used, and posteriorly corrected, by any filtering-based navigation/guidance stage deployed afterwards [4]. As explained by Thompson, Farrell, and Knight [5], the alignment requires the observation of, at least, two noncollinear vectors, whose components should be known in both body and navigation frames. Traditionally, the local gravity and Earth rate vectors, measured by stationary accelerometers and gyros, respectively, provided by an Inertial Measurement Unit (IMU), have been chosen for INS alignment purposes [6].

Despite the recognized efficiency of the aforementioned approach, it requires high quality (navigation-grade) gyros to be employed, which are expensive and contrast with the current expansion trend toward commercial low-cost navigation applications [7]. When low-cost IMUs are sought, a good candidate for replacing the Earth rate vector (for stationary alignment purposes) is the Earth magnetic flux density vector, which can be adequately observed by calibrated magnetometers [8]. When three-axial magnetometers are incorporated into an IMU, the resulting sensor set is often referred to as Attitude and Heading Reference System (AHRS) [9,10], or yet, Magnetic, Angular Rate, and Gravity (MARG) sensor array [11,12]. AHRSs have been successfully employed in a vast range of applications, including, but not limited to, Unmanned Aerial Vehicle (UAV) attitude control/stabilization [13], human body tracking [14,15], joint angle estimation [16], and mobile communications [17].

As in other INS-based integration approaches [1], AHRSs also resort to filtering schemes in order to have their inertial and magnetic sensor measurements optimally combined. The Extended Kalman filter (EKF) is probably the most employed integration architecture, wherein the attitude can be recursively propagated/updated based either on a linearized error model [1,18] or a nonlinear quaternion model [19,20]. Regardless of the adopted model, augmenting the states in order to estimate additional systematic/correlated noise contributions present in the sensor outputs is a recommended procedure [21]. Another modification that already proved to be effective consists in splitting the EKF in two separate filters, one devoted to tilt (pitch and roll) estimation and the other to that of heading [20,22]. Amongst the filtering-based AHRS integration architectures that do not employ EKFs (or its variants), stands out the Complementary Filter (CF), characterized by its efficient (although nonoptimal) operation and simplicity [23]. While much research effort has been dedicated to the investigation of these filtering-based AHRS integration architectures, only few works have studied the attitude initialization step, i.e., the alignment, that precedes any of them (either as a requirement for initializing the nonlinear attitude quaternion model or to guarantee the adequacy of using a small angle approximation for the attitude error states). As previously mentioned, the alignment is generally conducted in stationary conditions and is based on the observation of, at least, two noncollinear vectors. This paper, hence, is not aimed at investigating filtering-based attitude determination methods for AHRSs, but instead, it focuses on the particular problem of stationary AHRS alignment.

As recently analyzed by Fan, Li, and Liu [24], several attitude determination methods can be used to solve the AHRS stationary alignment problem. Among them, stands out the Three-Axis Attitude Determination (TRIAD)-based method, originally proposed by Black [25]. Despite being a straightforward analytical method, TRIAD has the drawback of only accommodating two vector observations per attitude computation. To solve this issue, the QUaternion ESTimator (QUEST) has been proposed by Shuster [26]. Based on Davenport’s work [27], QUEST optimally solved Wahba’s problem [28], becoming one of the most referred attitude determination methods so far [29]. When applied to the particular case of AHRS stationary alignment, however, QUEST proved to be unable to dissociate magnetometer errors from the estimation of pitch and roll [24,30,31]. A thorough investigation of the problem has not appeared in the literature afterwards. To overcome QUEST’s deficiency, Yun, Bachmann, and McGhee [32] proposed the Factored Quaternion Algorithm (FQA). Despite not being an optimal method, FQA demonstrated to be superior in accuracy (for roll and pitch), as well as 25% faster than QUEST. More recent, but still nonoptimal, alternatives to FQA are the Algebraic QUaternion Algorithm (AQUA) [11], the Super-fast Attitude of Accelerometer and Magnetometer (SAAM) [33], and the Simple-Structured Quaternion Estimator (SSQE) [34]. All of the latter claimed to reach the same accuracy as that of FQA but at an even lower computational cost.

Another attitude determination method, widely employed for AHRS stationary alignment purposes, is the Arc-TANgent (ATAN) solution [35]. Differently from TRIAD, QUEST, and FQA, which provide their attitude estimates in terms of Direction Cosine Matrices (DCM) and quaternions, respectively, ATAN calculates Euler angles directly, being hence susceptible to singularity problems [36]. Due to its simplicity, however, ATAN has been vastly used in the literature, both for the purpose of the AHRS stationary alignment [37,38,39,40], as for the attitude determination thereafter (i.e., in the navigation stage). In the latter case, ATAN’s attitude solution has been combined with solutions from different sensors/systems, both in a Complementary Filtering (CF) fashion [7,12,41,42] and Kalman Filtering (KF) fashion [19,43,44,45].

Even though the aforementioned AHRS stationary alignment approaches have been tested and validated in previous works, very little knowledge is available in the current literature about the analytic description of the residual errors remaining after the alignment or the benefits/drawbacks of each approach (from the analytical standpoint). In [46], for instance, a simplified overview of the main error sources affecting ATAN’s heading determination has been provided, but no mathematical background corroborated it. In [47], the analytical description of ATAN’s heading error has been proposed, as a function of the magnetometer (bias, scale factor, and cross-coupling) errors and the Earth magnetic model (declination angle) error. The drawbacks of the analysis, however, are: (a) it was only valid for two-axial (levelled) stationary AHRSs, and (b) the heading error equation was derived w.r.t. the magnetic heading, not to the true heading. Liu et al. [48] improved the aforementioned error analysis, by deriving a closed-form formula for ATAN’s heading error in a three-axial (nonlevelled) stationary AHRS application. Again, the analysis was confined to the magnetic heading error, which was equated as a function of the true pitch, roll and heading angles and not as a function of the magnetometer and Earth model parameter errors (which would have been more relevant). Even more simplified (or generic) analytical descriptions of ATAN’s (and QUEST’s) errors can be found in [1,29,44,49,50,51,52,53], which, however, do not provide much insight into the problem.

Apart from the above-mentioned works, most of the AHRS stationary alignment error analyses reported in the literature are solely based on numerical (simulated and/or experimental) results [54,55]. As an example, we may cite the work of Hu et al. [56], which numerically evaluated the impact of sensor cross-coupling and tilt errors in ATAN’s computed heading. Včelák et al. [57,58] improved Hu’s work, by also considering the detrimental effects of temperature variation. In [24,59,60,61,62], the authors numerically investigated the azimuth errors caused by changes in the magnetic field direction (i.e., in the declination and inclination angles), as well as variations (in the magnetic field magnitude) caused by common objects (i.e., hard-iron and soft-iron magnetisms). Sotak [38], Kuga and Carrara [30], and Del Rosario, Lovell and Redmond [42], in turn, evaluated the detrimental impact of accelerometer and magnetometer biases, as well as, scale factor errors, in ATAN, QUEST, and TRIAD, respectively. Bistrov [39], lastly, numerically analyzed the effect of sensor measurement noise in ATAN’s estimates. Even if the preceding numerical analyses are relevant, they are incomplete if not accompanied by a solid theoretical (analytical or stochastic) background. As a consequence, they can lead to misleading conclusions, as will be discussed throughout this paper.

To address the aforementioned issues, this paper presents an innovative and comprehensive analytical error description of four of the most employed attitude determination approaches for AHRS stationary alignment purposes, namely, TRIAD, QUEST, FQA, and ATAN. The main contribution of this paper is the derivation of novel closed-form formulae for the residual alignment errors developed in the corresponding DCM attitude solutions. For the purpose of the error analysis, errors in the accelerometers (constant biases), magnetometers (combined effect of constant biases and hard-iron magnetism), local gravity model, and Earth magnetic field model (flux density magnitude, declination angle, and inclination angle) are considered. Following the development of the closed-form formulae, a clarifying insight is provided into the AHRS stationary alignment problem, allowing us to compare the performance (in terms of ultimate accuracy) of the investigated algorithms properly, as well as to remove some misconceptions reported in previous works.

The remainder of this paper is organized as follows: Section 2 reviews TRIAD, QUEST, FQA, and ATAN algorithms. Section 3, in sequence, presents the procedures used to derive the novel closed-form formulae for the DCM residual errors, as well as the error analysis itself. Section 4 and Section 5 provide results from simulated and experimental tests, respectively, whilst Section 6 concludes the paper.

## 2. AHRS Alignment Formulations

This section reviews the most representative attitude determination methods employed for AHRS stationary alignment purposes. The discussion initiates with the Three-Axis Attitude Determination (TRIAD)-based method.

### 2.1. TRIAD Method

As briefly mentioned in Section 1, TRIAD is able to generate an estimate of the DCM relating body and navigation frames, from the observation of two arbitrary noncollinear vectors [25]. A suitable set of vectors for AHRS alignment purposes is the $g_{P}$ local plumb-bob gravity and the $m_{E}$ Earth magnetic flux density vectors. The analytical descriptions of each is straightforward in the navigation frame (In this paper, the navigation frame is represented by the superscript *l* and is defined with its $x_{l}$, $y_{l}$ and $z_{l}$ axes pointing to the North, East and Down (NED) directions, respectively.) [1],

(1) $$g_{P}^{l}={\left[\begin{array}{ccc} 0 & 0 & g_{P} \end{array}\right]}^{T}$$

(2) $$m_{E}^{l}=B{\left[\begin{array}{ccc} c\alpha c\gamma & s\alpha c\gamma & s\gamma \end{array}\right]}^{T}$$

where $g_{P}$ is the magnitude of the local plumb-bob gravity vector; *B* is the magnitude of the local Earth magnetic flux density vector; α and γ are the corresponding declination and inclination (dip) angles, respectively; and *s* and *c* stand for sine and cosine, respectively.

From (1) and (2), a third vector can be generated using the cross-product operator,

(3) $${\left(g_{P}\times m_{E}\right)}^{l}={\left[\begin{array}{ccc} -g_{P}Bs\alpha c\gamma & g_{P}Bc\alpha c\gamma & 0 \end{array}\right]}^{T}$$

Temporarily assuming that the AHRS is perfectly stationary and the accelerometers and magnetometers are uncorrupted, $g_{P}$, $m_{E}$ and $g_{P}\times m_{E}$ can be resolved in the body frame (In this paper, the body frame is represented by the superscript *b* and is defined with its $x_{b}$, $y_{b}$ and $z_{b}$ axes pointing forward, to the right-hand side and downward, all with respect to the vehicle on which the AHRS is mounted.) through the outputs of the sensors,

(4) $$g_{P}^{b}\approx -a_{SF}^{b}={\left[\begin{array}{ccc} -a_{x} & -a_{y} & -a_{z} \end{array}\right]}^{T}$$

(5) $$m_{E}^{b}\approx m_{m}^{b}={\left[\begin{array}{ccc} m_{x} & m_{y} & m_{z} \end{array}\right]}^{T}$$

(6) $${\left(g_{P}\times m_{E}\right)}^{b}\approx -{\left(a_{SF}\times m_{m}\right)}^{b}=\left[\begin{array}{c} a_{z}m_{y}-a_{y}m_{z}\\ a_{x}m_{z}-a_{z}m_{x}\\ a_{y}m_{x}-a_{x}m_{y} \end{array}\right]$$

where $a_{SF}$ is the specific force acceleration vector measured by the accelerometers; and $m_{m}$ is the total magnetic flux density vector measured by the magnetometers.

The $C_{l}^{b}$ DCM, relating body and navigation frames, can be equated as,

(7) $$𝓑=C_{l}^{b}𝓛$$

with,

(8) $$𝓑=\left[\begin{array}{ccc} g_{P}^{b} & m_{E}^{b} & {\left(g_{P}\times m_{E}\right)}^{b} \end{array}\right]$$

(9) $$𝓛=\left[\begin{array}{ccc} g_{P}^{l} & m_{E}^{l} & {\left(g_{P}\times m_{E}\right)}^{l} \end{array}\right]$$

From (7), it is obvious that,

(10) $$C_{b}^{l}={\left({𝓛}^{-1}\right)}^{T}{𝓑}^{T}$$

As (10) indicates, TRIAD’s attitude solution is directly given in terms of DCM and only requires elementary inverse/transpose operations over the 𝓑 and 𝓛 triads of vector observations.

### 2.2. QUEST Method

QUEST has been proposed by Shuster [26] aiming at solving Wahba’s problem [28]. As originally derived by Davenport [27], Wahba’s optimal solution (parametrized as a quaternion) consists of finding the normalized eigenvector corresponding to the largest eigenvalue of the following matrix,

(11) $$K=\left[\begin{array}{cc} S-\sigma I & Z\\ Z^{T} & \sigma \end{array}\right]$$

with,

(12) $$S=R+R^{T}$$

(13) σ=tr(R)

(14) $$Z={\left[\begin{array}{ccc} R_{23}-R_{32} & R_{31}-R_{13} & R_{12}-R_{21} \end{array}\right]}^{T}$$

(15) $$R=\sum w_{a}\left(u_{a}^{b}\cdot u_{a}^{l}\right)$$

where tr is the trace operator, $R_{ij}$ is the element of *R* in row *i* and column *j*, $u_{a}$ is an arbitrary unit vector, and $w_{a}$ is its associated weighting factor, subject to $\sum w_{a}=1$.

For AHRS stationary alignment purposes, suitable unit vectors are $u_{g}=g_{P}/\left|g_{P}\right|$ and $u_{m}=m_{E}/\left|m_{E}\right|$, which transform the latter into an optimal two vector observation attitude determination problem [63]. For this particular problem, Shuster [26] already has demonstrated that the $\lambda_{\max}$ maximum eigenvalue of (11) has a closed-form solution, which is given by,

(16) $$\lambda_{max}=\sqrt{w_{g}^{2}+2w_{g}w_{m}c\left(\theta_{g}-\theta_{m}\right)+w_{g}^{2}}$$

with,

(17) $$c\left(\theta_{g}-\theta_{m}\right)=\left(u_{g}^{l}\cdot u_{m}^{l}\right)\left(u_{g}^{b}\cdot u_{m}^{b}\right)+|u_{g}^{l}\times u_{m}^{l}\left|\right|u_{g}^{b}\times u_{m}^{b}|$$

Given $\lambda_{max}$, the computation of QUEST’s optimal quaternion (relating *l* and *b* frames) simplifies to [26],

(18) $$q_{l}^{b}=\frac{1}{\sqrt{1+{\left|p\right|}^{2}}}\left[\begin{array}{c} 1\\ p \end{array}\right]$$

with,

(19) $$p={\left[\left(\lambda_{max}+\sigma \right)I-S\right]}^{-1}Z$$

When QUEST’s optimal attitude is required in terms of DCM, it suffices doing [26],

(20) $$C_{b}^{l}=\left(q_{f}^{2}-q\cdot q\right)I+2qq^{T}+2q_{f}\left(q\times \right)$$

where $q_{f}$ is the first element of $q_{l}^{b}$, and (q×) is the skew-symmetric matrix representation of vector ***q***, formed from the three last elements of $q_{l}^{b}$.

### 2.3. FQA Method

The idea behind FQA is that a rigid body can be placed into any arbitrary attitude by performing three sequential rotations. In this sense, the AHRS stationary alignment problem can be broken into three pieces, each consisting on the computation of a rotation quaternion.

Let us consider, initially, the $q_{\theta }$ quaternion, which is relative to the pitch rotation. As suggested by Yun, Bachmann, and McGuee [32], $q_{\theta }$ can be computed as,

(21) $$q_{\theta }=c\left(\theta /2\right)\left[\begin{array}{cccc} 1 & 0 & 0 & 0 \end{array}\right]+s\left(\theta /2\right)\left[\begin{array}{cccc} 0 & 0 & 1 & 0 \end{array}\right]$$

with,

(22) $$s\left(\theta /2\right)=\mathrm{sign}\left(s\theta \right)\sqrt{(1-c\theta )/2}$$

(23) $$c\left(\theta /2\right)=\sqrt{(1+c\theta )/2}$$

where sign() is the signum function that returns +1 for positive arguments and −1 for negative arguments. To save computational effort, sθ and cθ do not need to be computed (in (22) and (23), respectively) using trigonometric functions, but instead,

(24) $$s\theta ={\bar{a}}_{x}$$

(25) $$c\theta =\sqrt{1-s^{2}\theta }$$

where,

(26) $${\left[\begin{array}{ccc} {\bar{a}}_{x} & {\bar{a}}_{y} & {\bar{a}}_{z} \end{array}\right]}^{T}=-u_{g}^{b}$$

Similar to the pitch quaternion, the $q_{\phi }$ quaternion, relative to the roll rotation, can be computed as [32],

(27) $$q_{\phi }=c\left(\phi /2\right)\left[\begin{array}{cccc} 1 & 0 & 0 & 0 \end{array}\right]+s\left(\phi /2\right)\left[\begin{array}{cccc} 0 & 1 & 0 & 0 \end{array}\right]$$

where s(ϕ/2) and c(ϕ/2) are calculated as in (22) and (23), respectively (obviously replacing θ by ϕ), with,

(28) $$s\phi =-{\bar{a}}_{y}/c\theta$$

(29) $$c\phi =-{\bar{a}}_{z}/c\theta$$

The $q_{\psi }$ heading quaternion, lastly, is computed as follows,

(30) $$q_{\psi }=c\left(\psi /2\right)\left[\begin{array}{cccc} 1 & 0 & 0 & 0 \end{array}\right]+s\left(\psi /2\right)\left[\begin{array}{cccc} 0 & 0 & 0 & 1 \end{array}\right]$$

Once again, s(ψ/2) and c(ψ/2) are calculated as in (22) and (23), respectively (replacing θ by ψ), with,

(31) $$\left[\begin{array}{c} c\psi \\ s\psi \end{array}\right]=\left[\begin{array}{cc} \;\;\;M_{x} & M_{y}\\ -M_{y} & M_{x} \end{array}\right]\left[\begin{array}{c} N_{x}\\ N_{y} \end{array}\right]$$

where $N={\left[N_{x}\;N_{y}\right]}^{T}$ is the normalized Earth magnetic flux density vector in the horizontal plane, and $M={\left[M_{x}\;M_{y}\right]}^{T}$ is the corresponding quantity measured by the magnetometers. As suggested by Yun, Bachmann, and McGuee [32], *N* and *M* can be calculated as follows,

(32) $$N=\frac{1}{\sqrt{m_{N}^{2}+m_{E}^{2}}}\left[\begin{array}{c} m_{N}\\ m_{E} \end{array}\right]$$

(33) $$M=\frac{1}{\sqrt{{}^{e}m_{x}^{2}{+}^{e}m_{y}^{2}}}\left[\begin{array}{c} {}^{e}m_{x}\\ {}^{e}m_{y} \end{array}\right]$$

where $m_{N}$ and $m_{E}$ are the north and east components of (2), respectively (In the rest of this paper, scalar variables accompanied by *N*, *E* and *D* subscripts indicate quantities in the north, east and down directions, respectively.); and ${}^{e}m_{x}$ and ${}^{e}m_{y}$ are second and third components of the ${}^{e}m_{m}$ intermediate Earth frame quaternion, computed as

(34) $${}^{e}m_{m}=q_{\theta }⊗q_{\phi }⊗{(}^{b}m_{m})⊗q_{\phi }^{-1}⊗q_{\theta }^{-1}$$

with,

(35) $${}^{b}m_{m}=\left[\begin{array}{cccc} 0 & m_{x} & m_{y} & m_{z} \end{array}\right]$$

where ⊗ represents the quaternion multiplication operator.

Once $q_{\theta }$, $q_{\phi }$ and $q_{\psi }$ have been computed, the $q_{b}^{l}$ final attitude quaternion relating body and navigation frames can be obtained by simply doing [32],

(36) $$q_{b}^{l}=q_{\theta }⊗q_{\phi }⊗q_{\psi }$$

When FQA’s attitude solution is required in terms of DCM, it suffices doing,

(37) $$C_{b}^{l}=\left[\begin{array}{ccc} q_{1}^{2}+q_{2}^{2}-q_{3}^{2}-q_{4}^{2} & 2(q_{2}q_{3}-q_{1}q_{4}) & 2(q_{2}q_{4}+q_{1}q_{3})\\ 2(q_{2}q_{3}+q_{1}q_{4}) & q_{1}^{2}-q_{2}^{2}+q_{3}^{2}-q_{4}^{2} & 2(q_{3}q_{4}-q_{1}q_{2})\\ 2(q_{2}q_{4}-q_{1}q_{3}) & 2(q_{3}q_{4}+q_{1}q_{2}) & q_{1}^{2}-q_{2}^{2}-q_{3}^{2}+q_{4}^{2} \end{array}\right]$$

where $q_{i}$ is the *i*-th element of $q_{b}^{l}$.

### 2.4. ATAN Method

Differently from the preceding methods, ATAN directly provides an estimate of the AHRS attitude through Euler angles. Basically, the rationale for the ϕ roll, θ pitch and ψ heading computation comes from the solution of the following equalities,

(38) $$g_{P}^{b}=C_{l}^{b}g_{P}^{l}$$

(39) $$m_{E}^{b}=C_{l}^{b}m_{E}^{l}$$

with $C_{l}^{b}$ defined as [64],

(40) $$C_{l}^{b}=\left[\begin{array}{ccc} c\theta c\psi & c\theta s\psi & -s\theta \\ -c\phi s\psi +s\phi s\theta c\psi & c\phi c\psi +s\phi s\theta s\psi & s\phi c\theta \\ s\phi s\psi +c\phi s\theta c\psi & -s\phi c\psi +c\phi s\theta s\psi & c\phi c\theta \end{array}\right]$$

Substitution of (1), (4) and (40) in (38), yields,

(41) $${\left[\begin{array}{ccc} a_{x} & a_{y} & a_{z} \end{array}\right]}^{T}={\left[\begin{array}{ccc} s\theta & -s\phi c\theta & -c\phi c\theta \end{array}\right]}^{T}g_{P}$$

whose solution, for ϕ and θ, is,

(42) $$\phi =\arctan_{2}\left(-a_{y}/-a_{z}\right)$$

(43) $$\theta =\arcsin(a_{x}/g_{P})$$

For the determination of the heading angle, (2), (5) and (40) can be substituted in (39), yielding, after rearrangement [1],

(44) $$\left[\begin{array}{c} m_{x}\\ m_{y}\\ m_{z} \end{array}\right]=\left[\begin{array}{ccc} c\theta & 0 & -s\theta \\ s\phi s\theta & -c\phi & s\phi c\theta \\ c\phi s\theta & s\phi & c\phi c\theta \end{array}\right]\left[\begin{array}{c} c\psi_{m}c\gamma \\ s\psi_{m}c\gamma \\ s\gamma \end{array}\right]B$$

with,

(45) $$\psi_{m}=\psi -\alpha$$

where $\psi_{m}$ is the magnetic heading, i.e., the heading w.r.t. the magnetic north. As derived in [1], the solution of (44), for $\psi_{m}$, is,

(46) $$\psi_{m}=\arctan_{2}\frac{-m_{y}c\phi +m_{z}s\phi }{m_{x}c\theta +m_{y}s\phi s\theta +m_{z}c\phi s\theta }$$

If (42) and (43) are further substituted in (46), one has, after simplification [65],

(47) $$\psi_{m}=\arctan_{2}\frac{g_{P}\left(a_{z}m_{y}-a_{y}m_{z}\right)}{a_{y}\left(a_{y}m_{x}-a_{x}m_{y}\right)-a_{z}\left(a_{x}m_{z}-a_{z}m_{x}\right)}$$

Once $\psi_{m}$ is obtained, and assuming α is known, ψ can be computed by direct substitution in (45). When ATAN’s DCM is required, it can be computed by substituting the previously obtained ϕ, θ, and ψ in the transposed version of (40).

## 3. Error Analysis

Regardless of the adopted approach, all AHRS stationary alignment algorithms presented in Section 2 rely on the same assumptions: vehicle perfectly stationary, sensors uncorrupted, and external information (local gravity and Earth magnetic field models) accurately known. Obviously, none of these assumptions is satisfied in real world conditions, which means that only a corrupted estimate of the true initial attitude can be obtained.

For the analysis that follows, let us assume that the true attitude is provided in terms of the $C_{b}^{l}$ DCM. Without loss of generality, we can equate the corrupted ${\hat{C}}_{b}^{l}$ to $C_{b}^{l}$ as [66],

(48) $${\hat{C}}_{b}^{l}=C_{b}^{l}+\delta C_{b}^{l}=\left(I+E\right)C_{b}^{l}$$

where *I* is the identity matrix, and $\delta C_{b}^{l}$ and *E* are two representations of ${\hat{C}}_{b}^{l}$ errors.

As derived in [66,67,68], the following relations hold, to first order in *E*,

(49) $$E=\delta C_{b}^{l}{\left(C_{b}^{l}\right)}^{T}={\hat{C}}_{b}^{l}{\left(C_{b}^{l}\right)}^{T}-I=E_{s}+E_{ss}$$

with,

(50) $$E_{s}=\frac{E+E^{T}}{2}=\frac{{\hat{C}}_{b}^{l}{\left({\hat{C}}_{b}^{l}\right)}^{T}-I}{2}=\left[\begin{array}{ccc} \eta_{N} & o_{D} & o_{E}\\ o_{D} & \eta_{E} & o_{N}\\ o_{E} & o_{N} & \eta_{D} \end{array}\right]$$

(51) $$E_{ss}=\frac{E-E^{T}}{2}=\left[\begin{array}{ccc} 0 & \varphi_{D} & -\varphi_{E}\\ -\varphi_{D} & 0 & \varphi_{N}\\ \varphi_{E} & -\varphi_{N} & 0 \end{array}\right]$$

where $\eta^{l}$, $o^{l}$, and $\varphi^{l}$ are the DCM normality, orthogonality, and alignment error vectors, respectively.

To develop an innovative and comprehensive error analysis for the AHRS stationary alignment formulations presented in Section 2, we can expand their true $C_{b}^{l}$ descriptions by linear perturbation technique,

(52) $$\begin{array}{cc} \delta C_{b}^{l}= & \frac{\partial C_{b}^{l}}{\partial a_{x}}\delta a_{x}+\frac{\partial C_{b}^{l}}{\partial a_{y}}\delta a_{y}+\frac{\partial C_{b}^{l}}{\partial a_{z}}\delta a_{z}+\frac{\partial C_{b}^{l}}{\partial m_{x}}\delta m_{x}+\frac{\partial C_{b}^{l}}{\partial m_{y}}\delta m_{y}\\ & +\frac{\partial C_{b}^{l}}{\partial m_{z}}\delta m_{z}+\frac{\partial C_{b}^{l}}{\partial g_{P}}\delta g_{P}+\frac{\partial C_{b}^{l}}{\partial B}\delta B+\frac{\partial C_{b}^{l}}{\partial \alpha }\delta \alpha +\frac{\partial C_{b}^{l}}{\partial \gamma }\delta \gamma \end{array}$$

where δx represents the error (Throughout this paper, and for the purpose of the error analysis herein presented, the errors in the investigated variables (sensor readings and gravity/Earth magnetic field model parameters) are assumed to be constant, i.e., systematic biases.) in the generic (scalar or matrix) variable *x*.

**Figure 1:**
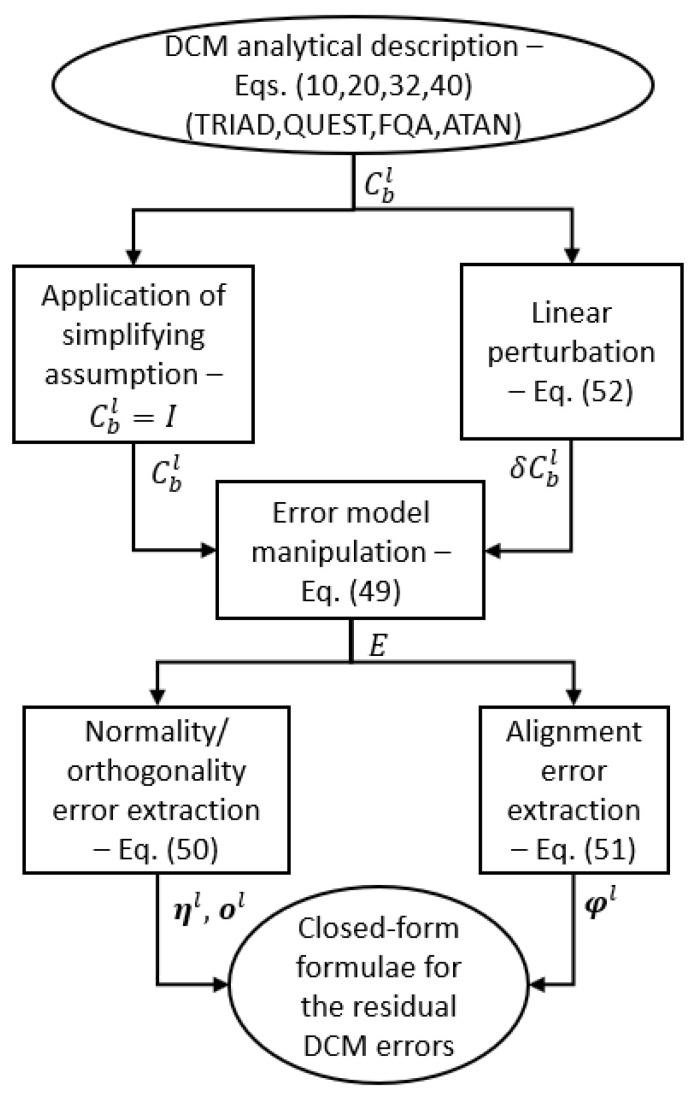
Summary of the closed-form formulae derivation process.

After analytically solving (52), for each alignment method, the result, substituted in (49), and then in (50) and (51), can be used to produce novel closed-form formulae for the DCM residual normality, orthogonality, and alignment errors. Unfortunately, for the purpose of the error analysis herein proposed, an arbitrary orientation of the body frame w.r.t. the navigation frame produces closed-form formulae which are overly complicated and not readily amenable to physical interpretation. For simplification purposes hence, hereinafter, we will assume that body and navigation frames are aligned. Figure 1 summarizes the main steps involved in the process of deriving the closed-form formulae for the residual DCM errors for each investigated AHRS stationary alignment method.

### 3.1. TRIAD Method

Solving (52) for TRIAD’s $C_{b}^{l}$, and substituting the result (jointly with (10)) in (49) to (51), yields the following closed-form formulae for the residual errors,

(53) $$\eta_{N}=\frac{c\alpha \;t\gamma }{g_{P}}\delta a_{x}+\frac{s\alpha \;t\gamma }{g_{P}}\delta a_{y}-\frac{s^{2}\alpha }{g_{P}}\delta a_{z}+\frac{c\alpha }{B\;c\gamma }\delta m_{x}+\frac{s\alpha }{Bc\gamma }\delta m_{y}-\frac{s^{2}\alpha }{g_{P}}\delta g_{P}+t\gamma \;\delta \gamma -\frac{1}{B}\delta B$$

(54) $$\eta_{E}=\frac{c\alpha \;t\gamma }{g_{P}}\delta a_{x}+\frac{s\alpha \;t\gamma }{g_{P}}\delta a_{y}-\frac{c^{2}\alpha }{g_{P}}\delta a_{z}+\frac{c\alpha }{B\;c\gamma }\delta m_{x}+\frac{s\alpha }{Bc\gamma }\delta m_{y}-\frac{c^{2}\alpha }{g_{P}}\delta g_{P}+t\gamma \;\delta \gamma -\frac{1}{B}\delta B$$

(55) $$\eta_{D}=-\frac{1}{g_{P}}\delta a_{z}-\frac{1}{g_{P}}\delta g_{P}$$

(56) $$o_{N}=-\frac{s(2\alpha )}{4g_{P}}\delta a_{x}-\frac{s^{2}\alpha }{2g_{P}}\delta a_{y}+\frac{s\alpha \;t\gamma }{2g_{P}}\delta a_{z}+\frac{s\alpha }{2B\;c\gamma }\delta m_{z}+\frac{s\alpha \;t\gamma }{2g_{P}}\delta g_{P}-\frac{s\alpha }{2}\delta \gamma -\frac{s\alpha \;t\gamma }{2B}\delta B$$

(57) $$o_{E}=-\frac{c^{2}\alpha }{2g_{P}}\delta a_{x}-\frac{s(2\alpha )}{4g_{P}}\delta a_{y}+\frac{c\alpha \;t\gamma }{2g_{P}}\delta a_{z}+\frac{c\alpha }{2B\;c\gamma }\delta m_{z}+\frac{c\alpha \;t\gamma }{2g_{P}}\delta g_{P}-\frac{c\alpha }{2}\delta \gamma -\frac{c\alpha \;t\gamma }{2B}\delta B$$

(58) $$o_{D}=\frac{s(2\alpha )}{2g_{P}}\delta a_{z}+\frac{s(2\alpha )}{2g_{P}}\delta g_{P}$$

(59) $$\varphi_{N}=-\frac{s(2\alpha )}{4g_{P}}\delta a_{x}+\frac{c^{2}\alpha +1}{2g_{P}}\delta a_{y}+\frac{s\alpha \;t\gamma }{2g_{P}}\delta a_{z}+\frac{s\alpha }{2B\;c\gamma }\delta m_{z}+\frac{s\alpha \;t\gamma }{2g_{P}}\delta g_{P}-\frac{s\alpha }{2}\delta \gamma -\frac{s\alpha \;t\gamma }{2B}\delta B$$

(60) $$\varphi_{E}=-\frac{s^{2}\alpha +1}{2g_{P}}\delta a_{x}+\frac{s(2\alpha )}{4g_{P}}\delta a_{y}-\frac{c\alpha \;t\gamma }{2g_{P}}\delta a_{z}-\frac{c\alpha }{2B\;c\gamma }\delta m_{z}-\frac{c\alpha \;t\gamma }{2g_{P}}\delta g_{P}+\frac{c\alpha }{2}\delta \gamma +\frac{c\alpha \;t\gamma }{2B}\delta B$$

(61) $$\varphi_{D}=-\frac{s\alpha \;t\gamma }{g_{P}}\delta a_{x}+\frac{c\alpha \;t\gamma }{g_{P}}\delta a_{y}-\frac{s\alpha }{B\;c\gamma }\delta m_{x}+\frac{c\alpha }{B\;c\gamma }\delta m_{y}-\delta \alpha$$

where *t* stands for tangent.

As (53) to (58) indicate, residual normality and orthogonality errors are developed in TRIAD’s DCM. According to Choukroun et al. [69], these errors are highly undesirable, as they collaborate to propagate errors in the vector transformation operations conducted at high computational rates (as it is the case in AHRSs). As (50) indicates, however, the normality and orthogonality errors can be estimated, to first order, uniquely from the computed ${\hat{C}}_{b}^{l}$, and hence, they are easily compensated in practical implementations [70,71]. Alternatively, a more interesting application for the normality and orthogonality errors consists in using their first order estimates to produce coarse (or pseudo) estimates for some of the sensor biases. As (53) to (58) clearly indicate, there is a linear relationship between the residual η normality and *o* orthogonality errors, and the underlying δa accelerometer and δm magnetometer biases corrupting the alignment. By using the former (normality/orthogonality errors) as a set of “pseudo"-measurements clearly casts the set of equations (53) to (58) into a least-squares framework, for which solutions can be estimated in terms of some of the sensor biases. A similar idea has been successfully used in [72] for estimating accelerometer and gyro biases, in [73] for accelerometer and magnetometer biases and in [74] for accelerometer and dual-antennae Global Navigation Satellite System (GNSS) baseline biases.

Unlike the normality and orthogonality errors, which can be estimated and compensated, the alignment errors cannot, and hence, are of greater concern. As suggested in [75], this is due to an observability deficiency of the AHRS stationary alignment, which prevents the latter from being properly estimated (and compensated), even in posterior Kalman-filter-based integration schemes. As (59) to (61) indicate, almost all of the investigated error sources contribute to the development of alignment errors in TRIAD’s ${\hat{C}}_{b}^{l}$. It is worth noting, however, that the error terms weighed by sα tends to be less detrimental, since, as analyzed by Chulliat et al. [76], the value of α rarely exceeds ±20 deg in terrestrial applications. Despite this, caution is advised when analyzing the provided equations. In (59), for instance, even though $\delta m_{z}$ is weighted by sα, its effect on $\varphi_{N}$ can be very significant, especially in the presence of hard-iron magnetism. As explained by Zhang and Yang [77], this magnetism is created by nearby man-made objects (including the navigational equipment itself) and is indistinguishable from the magnetometer bias. In the error analysis presented in this paper, hence, δm represents the cumulative effect of constant magnetometer biases and hard-iron magnetism.

When the related literature is consulted, it is found that (59) to (61) also bring relevant clarifications about TRIAD’s accuracy (for AHRS stationary alignment purposes). Equations (59) and (60), for instance, clearly demonstrate that the north and east alignment errors are corrupted not only by accelerometer errors, as claimed in [24,32] but also by magnetometer, gravity, and Earth magnetic field model errors. Equation (61), in sequence, demonstrates that the down alignment error is corrupted by both accelerometer and magnetometer errors, although the former are generally negligible, compared to the latter (i.e., $\delta a/g_{P}\ll \delta m/B$). None of these relations had been addressed in previous works [47,48]. Additionally, (61) provides conclusive evidence for the heading error behavior numerically reported in [59,60], according to which $\varphi_{D}$ seemed to vary, in magnitude, equal to the amount of the magnetic field deviation in the horizontal plane (δα).

Lastly, it is worth noting that the error terms weighted by tγ in the preceding formulae tend to be negligible at low latitudes. As the latitude increases, however, also does the value of γ, causing the residual alignment errors to be dramatically magnified (this is the main reason why the alignment is not practical near the Earth poles). Geometrically speaking, at the poles, the gravity and Earth magnetic flux density vectors become collinear, invalidating the fundamental assumption behind TRIAD.

### 3.2. QUEST Method

If an error analysis, similar to the one conducted for TRIAD, is developed for QUEST, i.e., if we apply (52) in (20) and substitute the result in (49) to (51), the following closed-form formulae arise for the DCM residual errors,

(62) $$\eta_{N}=\eta_{E}=\eta_{D}=o_{N}=o_{E}=o_{D}=0$$

(63) $$\begin{array}{c} \varphi_{N}=\frac{w_{g}}{g_{P}}\delta a_{y}-w_{m}[\frac{s(2\alpha )}{2g_{P}}\delta a_{x}-\frac{c^{2}\alpha }{g_{P}}\delta a_{y}\\ +\frac{s(2\alpha )s\gamma }{2B}\delta m_{x}+\frac{s^{2}\alpha s\gamma }{B}\delta m_{y}-\frac{s\alpha c\gamma }{B}\delta m_{z}+s\alpha \delta \gamma ] \end{array}$$

(64) $$\begin{array}{c} \varphi_{E}=-\frac{w_{g}}{g_{P}}\delta a_{x}+w_{m}[-\frac{s^{2}\alpha }{g_{P}}\delta a_{x}+\frac{s(2\alpha )}{2g_{P}}\delta a_{y}\\ +\frac{c^{2}\alpha s\gamma }{B}\delta m_{x}+\frac{s(2\alpha )s\gamma }{2B}\delta m_{y}-\frac{c\alpha c\gamma }{B}\delta m_{z}+c\alpha \delta \gamma ] \end{array}$$

(65) $$\varphi_{D}=-\frac{s\alpha \;t\gamma }{g_{P}}\delta a_{x}+\frac{c\alpha \;t\gamma }{g_{P}}\delta a_{y}-\frac{s\alpha }{B\;c\gamma }\delta m_{x}+\frac{c\alpha }{B\;c\gamma }\delta m_{y}-\delta \alpha$$

As (62) indicates, no normality and orthogonality errors are developed in QUEST’s DCM. This corroborates [67], which already had demonstrated that any quaternion parametrization of attitude, when converted to DCM, does not propagate normality and orthogonality errors (as long as the quaternion unit norm constraint is conserved). As previously mentioned, these errors are easily removed from the computed DCM, so, QUEST’s ability of not generating them is only significant from the computational efficiency standpoint. Equations (63) to (65), in turn, are more relevant, as they provide analytical proof to a behavior that, hitherto, had been only numerically demonstrated [32]. As (63) and (64) indicate (in comparison to (59) and (60)), QUEST’s north and east alignment errors are no longer corrupted by $\delta a_{z}$, $\delta g_{P}$ and δB error terms, but instead, by $\delta m_{x}$ and $\delta m_{y}$. As already explained, this may be very detrimental to be accuracy of $\varphi_{N}$ and $\varphi_{E}$, especially in the presence of hard-iron magnetism.

Another interesting evidence from (63) and (64) is: the detrimental impact of the magnetometer biases in $\varphi_{N}$ and $\varphi_{E}$ is basically dictated by $w_{m}$, which, as already defined, is the weight QUEST attributes to the magnetometer vector observations. As a general rule, the larger $w_{m}$, the worse (more biased) $\varphi_{N}$ and $\varphi_{E}$ will be. While this tells us that less biased estimates could be obtained by decreasing $w_{m}$, at the limit, where $w_{m}\to 0$, the attitude solution would become indeterminate, as only one vector ($u_{g}$) would remain being observed. These verifications are innovative and very enlightening, especially when compared to the results of other error analyses presented so far, whose results lack physical interpretation [29,53]. QUEST’s down alignment error, lastly, is expected to reach the same accuracy as that of TRIAD (see (61) and (65)), which corroborates the numerical results given in [32].

### 3.3. FQA/ATAN Methods

Closed-form formulae for the residual errors developed in FQA’s DCM can also be formulated. For this, it suffices applying (52) in (37), and substituting the result in (49) to (51). The outcomes are,

(66) $$\eta_{N}=\eta_{E}=\eta_{D}=o_{N}=o_{E}=o_{D}=0$$

(67) $$\varphi_{N}=\frac{1}{g_{P}}\delta a_{y}$$

(68) $$\varphi_{E}=-\frac{1}{g_{P}}\delta a_{x}$$

(69) $$\varphi_{D}=-\frac{s\alpha t\gamma }{g_{P}}\delta a_{x}+\frac{c\alpha t\gamma }{g_{P}}\delta a_{y}-\frac{s\alpha }{Bc\gamma }\delta m_{x}+\frac{c\alpha }{Bc\gamma }\delta m_{y}-\delta \alpha$$

As (66) indicates, no normality and orthogonality errors are developed in FQA’s DCM. Again, this corroborates theory [67], as FQA’s attitude solution is primarily given in terms of a quaternion. Equations (67) and (68), in turn, contrast to the conclusions of Yun, Bachmann, and McGuee [32], according to which FQA and TRIAD were said to have similar accuracies. As (67) to (69), in comparison to (59) and (61), clearly indicate, this is only true for $\varphi_{D}$, as FQA’s $\varphi_{N}$ and $\varphi_{E}$ are not corrupted by magnetometer errors, neither by variations in the Earth magnetic field model. Another interesting verification is that FAQ’s $\varphi_{N}$ and $\varphi_{E}$ accuracies are similar to those of QUEST, for the particular scenario of $w_{m}=0$. As this would result in an indeterminate solution for QUEST, is it straightforward to conclude therefore that FQA’s accuracy is globally superior to that of QUEST (and ergo, of TRIAD).

Finally, closed-form formulae for ATAN’s DCM residual errors can be also derived. In this case, (42), (43), (45), and (47) are substituted in (40) and the result, used for solving (52). After substitution in (49) and (51), the conclusions are: ATAN’s error equations are exactly the same as those derived for FQA (for brevity, therefore, they are not repeated here). This verification confirms the results numerically obtained in [42], according to which ATAN’s north and east alignment errors proved not to be corrupted by magnetometer errors, neither by variations in the Earth magnetic field model. Since ATAN provides the same accuracy as FQA, which is, by inspection, better than TRIAD and QUEST, also ATAN is superior to the latter. From the computational efficiency standpoint, however, FQA can still be considered superior to ATAN, since, as suggested in [32], it avoids the explicit computation of trigonometric functions.

## 4. Simulation Results

To evaluate the innovative error analysis presented in Section 3, as well as the outlined verifications, a simulated test was carried out. For the purpose of the test, accelerometer and magnetometer data were generated at 100 Hz, considering the ideal scenario of body and navigation frames perfectly aligned. The accelerometers and magnetometers were purposely corrupted by 5 mg and 5 mG of constant biases, as well as by white random noises with root Power Spectral Densities (PSDs) of 0.1 mg/$\sqrt{\mathrm{Hz}}$ and 0.2 mG/$\sqrt{\mathrm{Hz}}$, respectively. These values were chosen as they correspond to typical error parameters of automotive-grade AHRSs found in manufacturer data-sheets (see Section 5) and textbooks [1]. The sensors were simulated at latitude −23.2131 deg, longitude −45.8606 deg, and altitude 629 m. The local gravity acceleration and Earth magnetic field were calculated according to the models described in [76,78], respectively. Corresponding errors (constant biases) of 0.005 mg, 0.1 mG, 0.1 deg, and 0.1 deg were purposely added in the values of gravity, Earth magnetic flux density magnitude, declination angle, and inclination angle, respectively.

**Figure 2:**
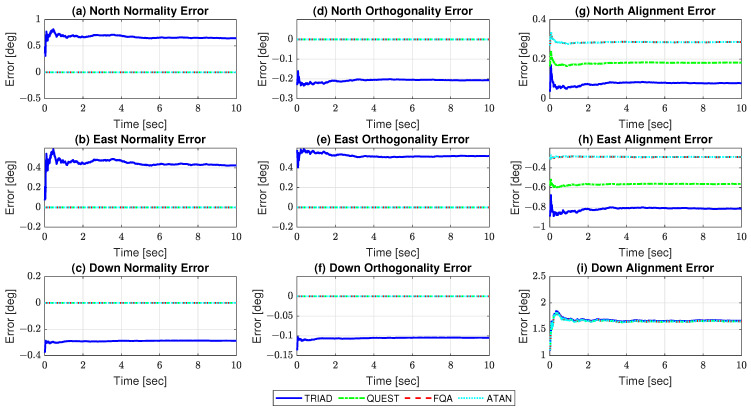
Estimated Direction Cosine Matrices (DCM) residual errors in simulated test.

For the purpose of the test, we compared TRIAD, QUEST, FQA, and ATAN, in terms of the achieved AHRS stationry alignment accuracies. For QUEST, particularly, values of $w_{g}=0.75$ and $w_{m}=0.25$ were chosen, following the methodology suggested in [79,80]. Based on the simulated values for the sensor, gravity, and Earth magnetic field errors, the closed-form formulae derived in Section 3 were used to generate predictions for the DCM residual normality, orthogonality, and alignment errors (Table 1). Figure 2 depicts the estimated errors as function of time, where a continuous averaging of the sensor outputs has been adopted, in order to smooth the noise. Table 2 summarizes the errors estimated at the completion of the simulated test, as well as the associated standard uncertainties (The standard uncertainties represent the coverage intervals within which the true values of the estimates are expected to lie (with a level of confidence of 95%, i.e., coverage factor of 2 [81]). In this paper, the standard uncertainties have been computed by dividing the standard deviations of the estimates (characterizing their dispersion about their mean values) by the square root of the number of observations. According to [81], this corresponds to the Type A evaluation of standard uncertainty, i.e., standard uncertainties obtained experimentally, by the statistical analysis of series of observations.).

As can be seen, the estimated errors nicely agree with the predicted ones (confidence intervals experimentally obtained in Table 2 encompass the predicted values in Table 1), which validates the proposed error analysis. Figure 2a–f, in particular, indicate that only TRIAD develops normality and orthogonality errors in the computed DCM (as expected). Figure 2g,h, in turn, demonstrate that, while FQA’s and ATAN’s north and east alignment errors are only corrupted by accelerometer errors, those from TRIAD and QUEST also depend on magnetometer biases, as well as on gravity and Earth magnetic field model errors. In the specific case of QUEST, the north and east alignment errors are found to be further dependent on $w_{m}$. Lastly, Figure 2i evidences that, as predicted, no difference exists, in terms of accuracy, between the down alignment error developed in TRIAD’s, QUEST’s, FQA’s, and ATAN’s computed DCM.

Regarding the standard uncertainties of the estimated DCM errors, Table 2 clearly shows that larger (i.e., more uncertain) values are expected to exist for errors that depend on magnetometer readings (see $\varphi_{N}$ and $\varphi_{E}$ for TRIAD and QUEST, for instance). A statistical and more appropriate way of characterizing the dispersions (i.e., the standard uncertainties) of the estimated DCM errors around their predicted values is using a Monte Carlo simulation. To perform the latter, 10,000 statistically independent simulated runs (of ten seconds each) were repeated (This number of Monte Carlo runs has been chosen based on a trade-off between its ability of adequately recuperating the Probability Density Function (PDF) of the simulated noise and the computational effort. It is in agreement with previous works published in the area [19,43,50,82,83,84], as well as with [85].), whereas the main input parameters for the simulation were allowed to vary (randomly) according to the standard deviations summarized in Table 3. The predicted and estimated DCM errors obtained at the completion of each run were saved and subtracted from one another. The mean values and standard uncertainties of the formed differences (deviations) are summarized in Table 4.

As can be seen, the mean values of all DCM error deviations (estimated minus predicted values) are close zero, indicating that the closed-form formulae derived in Section 3 are statically reliable (unbiased) estimators of the current DCM residual errors. The obtained standard uncertainties, in turn, follow the same pattern evidenced for the standard uncertainties of Table 2, i.e., closed-form formulae that are independent on magnetometer errors are expected to be less uncertain.

## 5. Experimental Results

**Figure 3:**
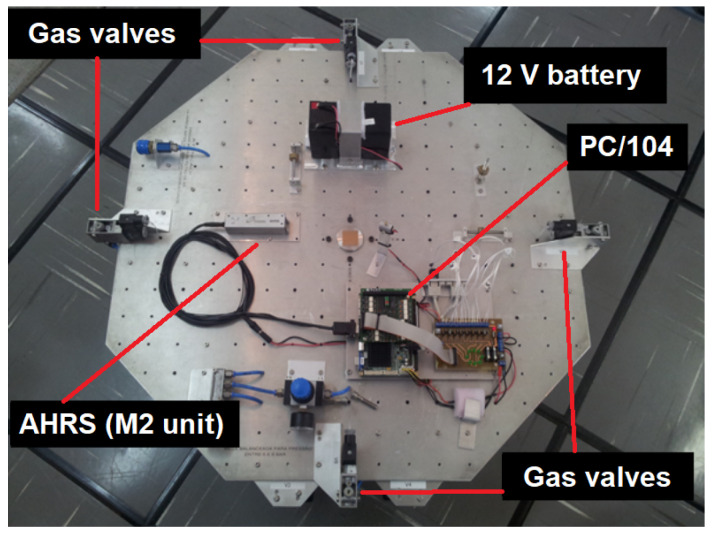
Semispherical 3-DOF air bearing, available at INPE.

To verify the results achieved in the simulated tests, an experimental test was conducted. The employed AHRS, namely, an automotive-grade M2 unit from Innalabs^®^, was mounted aligned to the navigation frame on a semispherical three degrees of freedom (3-DOF) air bearing [86], as depicted in Figure 3. The test was conducted at the Brazilian National Institute for Space Research (INPE), in São José dos Campos, Brazil. The geographic coordinates of the site are: −23.2113 deg of latitude, −45.1408 of longitude, and 641 m of altitude.

To compensate the sensor data (magnetometers, in particular) for residual scale factor/cross-coupling errors and soft-iron magnetism, which are not accounted for in the error analysis presented in this paper, the latter were precalibrated using the algorithm proposed in [87]. In sequence, a stationary sample of the precalibrated data was continuously averaged over about ten seconds (producing ${\bar{a}}_{SF}^{b}$ and ${\bar{m}}_{m}^{b}$), and estimates for the ${\hat{g}}_{P}^{l}$ local gravity and ${\hat{m}}_{E}^{l}$ Earth magnetic flux density vectors were computed (using the models described in [78,88], respectively). Since, in the concerned test, the AHRS was mounted aligned to the navigation frame, the following relations held (The accuracy in these approximations is basically limited to the accuracy of the semispherical 3-DOF air bearing rotating mechanisms [86].): ${\bar{a}}_{SF}^{b}\approx -{\hat{g}}_{P}^{l}$ and ${\bar{m}}_{m}^{b}\approx {\hat{m}}_{E}^{l}$. Thus, by numerically subtracting $-{\hat{g}}_{P}^{l}$ and ${\hat{m}}_{E}^{l}$ from the calibrated ${\bar{a}}_{SF}^{b}$ and ${\bar{m}}_{m}^{b}$, respectively, coarse estimates for the residual constant biases corrupting the outputs of the accelerometers and magnetometers were obtained, namely,

(70) $$\delta {\hat{a}}_{SF}^{b}={\left[\begin{array}{ccc} -0.4736 & -1.5073 & -1.8667 \end{array}\right]}^{T}\mathrm{mg}$$

(71) $$\delta {\hat{m}}_{m}^{b}={\left[\begin{array}{ccc} 10.5987 & 2.5724 & -4.1518 \end{array}\right]}^{T}\mathrm{mG}$$

**Figure 4:**
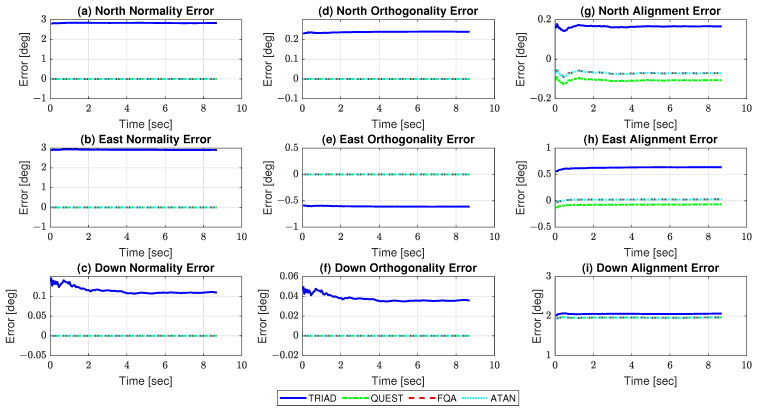
Estimated DCM residual errors in experimental test.

By substituting (70) and (71) into the closed-form formulae derived in Section 3 and also (purposely) considering the existence of errors (constant biases) of 0.005 mg, 0.1 mG, 0.1 deg, and 0.1 deg, in the values of gravity, and Earth magnetic flux density magnitude, declination angle, and inclination angle, respectively, predictions were obtained for the DCM residual errors in the experimental test (Table 5). Figure 4 presents the estimated errors as function of time, while Table 6 summarizes the errors at the completion of the experimental test alongside their standard uncertainties.

As can be noticed, the estimated errors corroborate the predicted ones, following the same pattern evidenced in the single-run simulated test. Differently from Section 4, we verify now that the confidence intervals fail to encompass all the predicted values (in general, by a very small amount). This, however, is consistent, as the predicted DCM errors were not computed based on the true (and in this case, unknown) values of the sensor biases (as done in Section 4) but instead only on the coarse estimates of (70) and (71). These results, hence, confirm the validity of the innovative error analysis proposed in this paper, as well as the outlined verifications about TRIAD’s, QUEST’s, FQA’s, and ATAN’s accuracies for AHRS stationary alignment purposes.

## 6. Conclusions

In this paper, four well established attitude determination algorithms for Attitude and Heading Reference Systems (AHRSs) stationary alignment have been reviewed, namely, the Three-Axis Attitude Determination (TRIAD)-based method, the QUaternion ESTimator (QUEST), the Factored Quaternion Algorithm (FQA), and the Arc-TANgent (ATAN) solution. A detailed and innovative error analysis has been proposed, considering the ideal assumption of body and navigation frames perfectly aligned. For the purpose of the latter, we considered errors in the accelerometer/magnetometer readings (cumulative effect of constant biases and hard-iron magnetism) and in the gravity/Earth magnetic field models (flux density magnitude, declination angle, and inclination angle). As the main contribution of this study, novel closed-form formulae for the Direction Cosine Matrix (DCM) residual errors (normality, orthogonality, and alignment errors) were analytically derived. These formulae brought analytical insight into the problem, allowing us to compare the performance of the investigated alignment formulations (in terms of ultimate accuracy) as well as to remove some misconceptions reported in previous works.

As main conclusions of this investigation, we may summarize: (a) TRIAD is the sole algorithm producing normality and orthogonality errors in the computed DCM; (b) FQA’s and ATAN’s north and east alignment errors are uniquely caused by *y*- and *x*-axis accelerometer biases, respectively; (c) larger north and east alignment errors are expected to exist when using TRIAD and QUEST, as the latter are also function of the magnetometer biases, the inclination angle error, and the error in the Earth magnetic flux density magnitude; (d) in the specific case of QUEST, the detrimental effect of magnetometer biases in the north and east alignment errors are weighted by $w_{m}$; (e) the DCM down alignment error is the same for all investigated approaches and is mainly caused by *x*- and *y*-axis magnetometer biases, as well as the declination angle error.

As suggestions for future work, the authors plan to expand their error analysis for the generic case of body and navigation frames arbitrarily oriented, which is more relevant for practical implementations. In this sense, it is found that a procedure similar to the one presented in [72,89] can be adopted. Moreover, the consideration of scale factor/cross-coupling errors (which would include misalignments and soft-iron effects for the magnetometers) also seems a topic worthy of future investigation.

## Figures and Tables

**Table 1:** Predicted DCM normality, orthogonality, and alignment errors in simulated test.

Errors	TRIAD	QUEST	FQA/ATAN
$\eta_{N}$ [deg]	0.6163	0	0
$\eta_{E}$ [deg]	0.4084	0	0
$\eta_{D}$ [deg]	−0.2874	0	0
$o_{N}$ [deg]	−0.2091	0	0
$o_{E}$ [deg]	0.5224	0	0
$o_{D}$ [deg]	−0.0992	0	0
$\varphi_{N}$ [deg]	0.0779	0.1802	0.2871
$\varphi_{E}$ [deg]	−0.8095	−0.5542	−0.2871
$\varphi_{D}$ [deg]	1.6754	1.6754	1.6754

**Table 2:** Estimated DCM normality, orthogonality, and alignment errors at the completion of the simulated test and associated standard uncertainties.

Errors	TRIAD	QUEST	FQA	ATAN
$\eta_{N}$ [deg]	0.6451	0.0000	0.0000	0.0000
	±0.0405	±0.0000	±0.0000	±0.0000
$\eta_{E}$ [deg]	0.4235	0.0000	0.0000	0.0000
	±0.0401	±0.0000	±0.0000	±0.0000
$\eta_{D}$ [deg]	−0.2873	0.0000	0.0000	0.0000
	±0.0036	±0.0000	±0.0000	±0.0000
$o_{N}$ [deg]	−0.2070	0.0000	0.0000	0.0000
	±0.0076	±0.0000	±0.0000	±0.0000
$o_{E}$ [deg]	0.5197	0.0000	0.0000	0.0000
	±0.0190	±0.0000	±0.0000	±0.0000
$o_{D}$ [deg]	−0.1049	0.0000	0.0000	0.0000
	±0.0013	±0.0000	±0.0000	±0.0000
$\varphi_{N}$ [deg]	0.0782	0.1824	0.2862	0.2862
	±0.0084	±0.0045	±0.0036	±0.0036
$\varphi_{E}$ [deg]	−0.8140	−0.5630	−0.2911	−0.2896
	±0.0191	±0.0079	±0.0037	±0.0036
$\varphi_{D}$ [deg]	1.6595	1.6471	1.6481	1.6484
	±0.0391	±0.0387	±0.0387	±0.0387

**Table 3:** Standard deviations of Monte Carlo simulated test main input parameters.

Parameter	Standard Deviation
Latitude (δL) [deg]	30
Longitude (δλ) [deg]	60
Altitude (δh) [m]	1000
Accelerometer biases ($\delta a_{x}$, $\delta a_{y}$, $\delta a_{z}$) [mg]	1
Magnetometer biases ($\delta m_{x}$, $\delta m_{y}$, $\delta m_{z}$) [mG]	5
Gravity magnitude ($\delta g_{P}$) [mg]	0.005
Earth’s magnetic flux density magnitude (δB) [mG]	0.1
Earth’s magnetic flux density inclination/declination angles (δα, δγ) [deg]	0.1

**Table 4:** Mean values and standard uncertainties of DCM normality, orthogonality, and alignment error deviations (estimated minus predicted values) in Monte Carlo simulated test.

Error Deviations	TRIAD	QUEST	FQA	ATAN
$\delta \eta_{N}$ [deg]	0.0607	0.0000	0.0000	0.0000
	±0.0048	±0.0000	±0.0000	±0.0000
$\delta \eta_{E}$ [deg]	0.0462	0.0000	0.0000	0.0000
	±0.0048	±0.0000	±0.0000	±0.0000
$\delta \eta_{D}$ [deg]	0.0001	0.0000	0.0000	0.0000
	±0.0000	±0.0000	±0.0000	±0.0000
$\delta o_{N}$ [deg]	−0.0001	0.0000	0.0000	0.0000
	±0.0001	±0.0000	±0.0000	±0.0000
$\delta o_{E}$ [deg]	−0.0001	0.0000	0.0000	0.0000
	±0.0002	±0.0000	±0.0000	±0.0000
$\delta o_{D}$ [deg]	−0.0038	0.0000	0.0000	0.0000
	±0.0007	±0.0000	±0.0000	±0.0000
$\delta \varphi_{N}$ [deg]	−0.0001	−0.0004	0.0000	0.0000
	±0.0001	±0.0001	±0.0000	±0.0001
$\delta \varphi_{E}$ [deg]	0.0001	−0.0006	0.0000	0.0000
	±0.0002	±0.0001	±0.0000	±0.0001
$\delta \varphi_{D}$ [deg]	0.0000	−0.0010	−0.0010	0.0018
	±0.0004	±0.0020	±0.0020	±0.0039

**Table 5:** Predicted DCM normality, orthogonality, and alignment errors in experimental test.

Errors	TRIAD	QUEST	FQA/ATAN
$\eta_{N}$ [deg]	2.7353	0	0
$\eta_{E}$ [deg]	2.8126	0	0
$\eta_{D}$ [deg]	0.1069	0	0
$o_{N}$ [deg]	0.2463	0	0
$o_{E}$ [deg]	−0.6151	0	0
$o_{D}$ [deg]	0.0369	0	0
$\varphi_{N}$ [deg]	0.1597	−0.1290	−0.0865
$\varphi_{E}$ [deg]	0.6423	−0.0790	0.0272
$\varphi_{D}$ [deg]	1.9875	1.9875	1.9875

**Table 6:** Estimated DCM normality, orthogonality, and alignment errors at the completion of the experimental test and associated standard uncertainties.

Errors	TRIAD	QUEST	FQA	ATAN
$\eta_{N}$ [deg]	2.8309	0.0000	0.0000	0.0000
	±0.0064	±0.0000	±0.0000	±0.0000
$\eta_{E}$ [deg]	2.9083	0.0000	0.0000	0.0000
	±0.0094	±0.0000	±0.0000	±0.0000
$\eta_{D}$ [deg]	0.1097	0.0000	0.0000	0.0000
	±0.0072	±0.0000	±0.0000	±0.0000
$o_{N}$ [deg]	0.2387	0.0000	0.0000	0.0000
	±0.0016	±0.0000	±0.0000	±0.0000
$o_{E}$ [deg]	−0.6095	0.0000	0.0000	0.0000
	±0.0042	±0.0000	±0.0000	±0.0000
$o_{D}$ [deg]	0.0358	0.0000	0.0000	0.0000
	±0.0027	±0.0000	±0.0000	±0.0000
$\varphi_{N}$ [deg]	0.1661	−0.1065	−0.0704	−0.0704
	±0.0065	±0.0065	±0.0068	±0.0068
$\varphi_{E}$ [deg]	0.6384	−0.0679	0.0292	0.0293
	±0.0049	±0.0050	±0.0063	±0.0063
$\varphi_{D}$ [deg]	2.0622	1.9672	1.9672	1.9672
	±0.0061	±0.0059	±0.0059	±0.0059
